# Association between sarcopenia and clinical outcomes in patients with hepatocellular carcinoma: an updated meta-analysis

**DOI:** 10.1038/s41598-022-27238-z

**Published:** 2023-01-17

**Authors:** Yusheng Guo, Yanqiao Ren, Licheng Zhu, Lian Yang, Chuansheng Zheng

**Affiliations:** 1 Department of Radiology, Union Hospital, Tongji Medical College, Huazhong University of Science and Technology, No. 1277 Jiefang Avenue, Wuhan, 430022 China; 2 Hubei Key Laboratory of Molecular Imaging, Wuhan, 430022 China

**Keywords:** Cancer, Computational biology and bioinformatics

## Abstract

Although numerous studies have reported the association between sarcopenia and the prognosis of hepatocellular carcinoma (HCC) patients, there is lack of a newer and more comprehensive meta-analysis. Herein, a comprehensive literature search was performed on PubMed, Web of Science, the Cochrane Library, and Embase databases to identify relevant studies published up to February 2022. The outcomes were overall survival (OS), recurrence, progression‐free survival, tumor response, severe postoperative complications, and toxicity of drugs. A total of 57 studies involving 9790 HCC patients were included in the meta-analysis. The pooled prevalence of sarcopenia in HCC patients was 41.7% (95% CI 36.2–47.2%). Results demonstrated that sarcopenia was significantly associated with impaired OS (HR: 1.93, 95% CI 1.73–2.17, *P* < 0.001), higher risk of tumor recurrence (HR: 1.75, 95% CI 1.56–1.96, *P* < 0.001), lower objective response rate (OR: 0.37 95% CI 0.17–0.81, *P* = 0.012), and more drug-related adverse events (OR: 2.23, 95% CI 1.17–4.28, *P* = 0.015) in HCC patients. The subgroup analyses revealed that the OS of patients at the early stage of tumor was more severely affected by sarcopenia than for patients at other stages. Moreover, the presence of cirrhosis and Child Pugh class B increased the hazard of mortality from sarcopenia. This study has shown that sarcopenia is highly associated with poor prognosis in HCC patients. In addition, cirrhosis and poor liver functional reserve increase the danger of sarcopenia. OS was more impaired in HCC patients with sarcopenia at early stage of tumor than at other tumor stages.

## Introduction

Hepatocellular carcinoma (HCC) is the sixth most common cancer and the fourth leading cause of cancer-related death^1^. Studies have reported that HCC is closely associated with chronic liver disease and cirrhotic livers^2^, and patients with these comorbidities commonly suffer poor appetite, malabsorption, and anorexia, which ultimately leads to the abnormality of nutritional status and skeletal muscle condition^3^. Although there are some methods that can predict the prognosis of patients according to tumor stage, such as Barcelona Clinic Liver Cancer (BCLC) staging classification and tumor-node-metastasis (TNM) classification^4,5^, these methods do not evaluate the nutritional status and skeletal muscle condition. In addition, despite the Child–Pugh score including the indicators of albumin and ascites which reflect the nutritional status to some degree, it is still limited by its inherent subjectivity in the assessment of Child–Pugh^6^. Moreover, although an objective laboratory parameter is used in the Albumin-bilirubin (ALBI) score^7^, it is hard to comprehensively assess the nutritional status using albumin alone. Therefore, this calls for studies to develop a new method that comprehensively reflects the nutritional status and skeletal muscle status of patients.

Sarcopenia is a skeletal muscle disorder characterized by progressive loss of skeletal muscle mass and strength^8–10^. Studies have proved that reduced muscle mass is associated with decreased immunity, reduced quality of life, and higher prevalence of fractures or falls, which eventually results in poor clinical outcomes^11–13^. In recent years, an increasing number of studies have reported that the loss of skeletal muscle mass is associated with poor prognoses in cancer patients, including HCC^14–16^. However, to date, there is no large prospective study which has explored the relationship between sarcopenia and HCC. A recent meta-analysis indicated that sarcopenia impairs clinical outcomes in patients with cirrhosis^6^. However, although the study included a certain number of HCC patients, it still proved inadequate in determining the association between sarcopenia and HCC. In addition, the meta-analyses on sarcopenia and HCC published before 2019 included very few patients and studies, which limited their statistical power for subgroup analyses or clinical outcomes^17,18^.

Therefore, there is need for a newer and comprehensive meta-analysis to evaluate the influence of sarcopenia on prognosis of HCC patients, with more detailed subgroup analyses, larger sample sizes, and more clinical outcomes, such as overall survival (OS), recurrence, tumor response, and adverse events. This study investigated a large number of patients and conducted subgroup analyses of different clinical outcomes, with the overarching goal of exploring the association between sarcopenia and HCC.

## Methods

### Search strategy

This meta-analysis was performed in accordance with the PRISMA guidelines^19^ and the protocol for this meta‐analysis was available in PROSPERO (CRD42022310433). A comprehensive search was performed on PubMed, Web of Science, the Cochrane Library, and Embase databases to identify relevant studies published up to February 2022. The following key words were used: “sarcopenia”, “sarcopenic”, “skeletal muscle”, “muscle atrophy”, “muscle wasting”, “muscular depletion “, “HCC”, “liver cancer”, “liver neoplasm”, and “hepatocellular carcinoma”. In addition, the references of included studies were manually scanned to retrieve potentially missing studies.

### Inclusion and exclusion criteria

Two independent authors (Yusheng Guo and Yanqiao Ren) conducted the preliminary review of literature identified in the databases by reading titles and abstracts. Studies were considered eligible if they met the following inclusion criteria: (1) were limited to English articles; (2) evaluated the impact of sarcopenia in HCC patients; (3) reported OS, disease-free survival (DFS), recurrence-free survival (RFS), objective response rate (ORR), disease control rate (DCR), toxicity of drugs, or postoperative complications were reported. In instances where multiple publications reported overlapping data, the study with the largest sample size was considered. Exclusion Criteria: (1) Comments, editorials, letters, case reports, reviews, and meta-analyses were not considered. (2) Duplicate documents were deleted.

### Data extraction

Two authors independently extracted the following data from the included studies: year of publication, name of first author, region, treatment mean, diagnostic method, cut‐off value, HCC stage, outcomes, number of enrolled patients, number of patients with sarcopenia, and sex ratio. Each study was independently assessed by the two authors using the Newcastle–Ottawa scale (NOS)^20^, and studies with NOS score ≥ 6 were considered high‐quality studies. Any disagreements were resolved by discussion or consensus with a third author (Lian Yang or Chuansheng Zheng).

### Statistical analyses

All statistical analyses were performed using R software (version 4.1.0). Before conducting the meta-analysis, a heterogeneity test was performed using χ2 tests (α = 0.10) and the I2 metric. *P* < 0.05 indicated the existence of heterogeneity, and studies with I^2^ > 35% were considered as having high heterogeneity. Notably, a random effects model (high heterogeneity) or a fixed effects model (low heterogeneity) was used to pool data for meta-analysis. Next, a forest map was drawn, and the HR or OR and its 95% confidence interval (CI) were described and discussed. Possible sources of heterogeneity were determined using Baujat plots and sensitivity analyses were then conducted through sequential omission of studies. Subgroup analyses of OS, recurrence, and tumor response were performed based on patients’ characteristics. Finally, Egger’s tests and funnel plot were performed to evaluate publication biases. Two-sided *P* < 0.05 were considered statistically significant for all statistical procedures.

## Results

**Figure 1:**
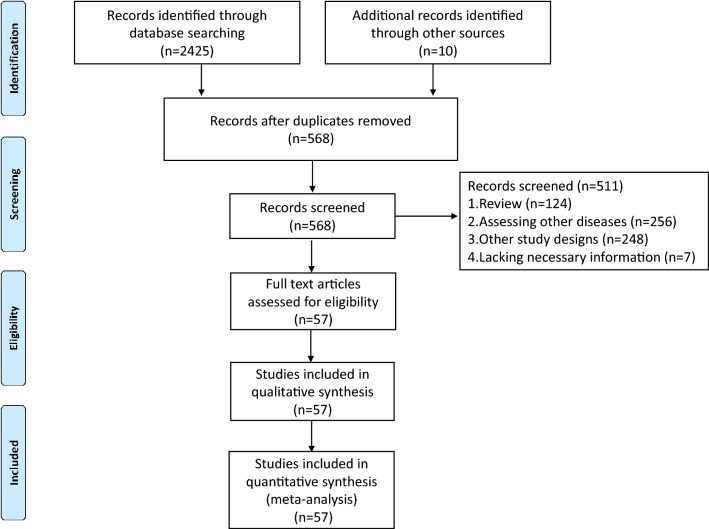
Flow diagram of study selection for inclusion in this meta-analysis.

A total of 2435 studies were identified after screening the databases, from which 1867 studies were excluded, followed by reviewing the abstracts of 568 studies in accordance with the inclusion criteria. Finally, 57 studies^3,21–76^ were included in this meta-analysis after detailed full-text examination (Fig. 1). Notably, sarcopenia was defined based on computed tomography (CT) or magnetic resonance imaging (MRI) in all enrolled studies. Given that two studies by Saeki et al.^46,56^ had duplicated the patients, the study with more patients was included in OS and subgroup analyses^56^. Saeki et al.^46^ was only used to explore the prevalence of sarcopenia. All enrolled studies were retrospective in design.

### Characteristics of included studies

**Figure 2:**
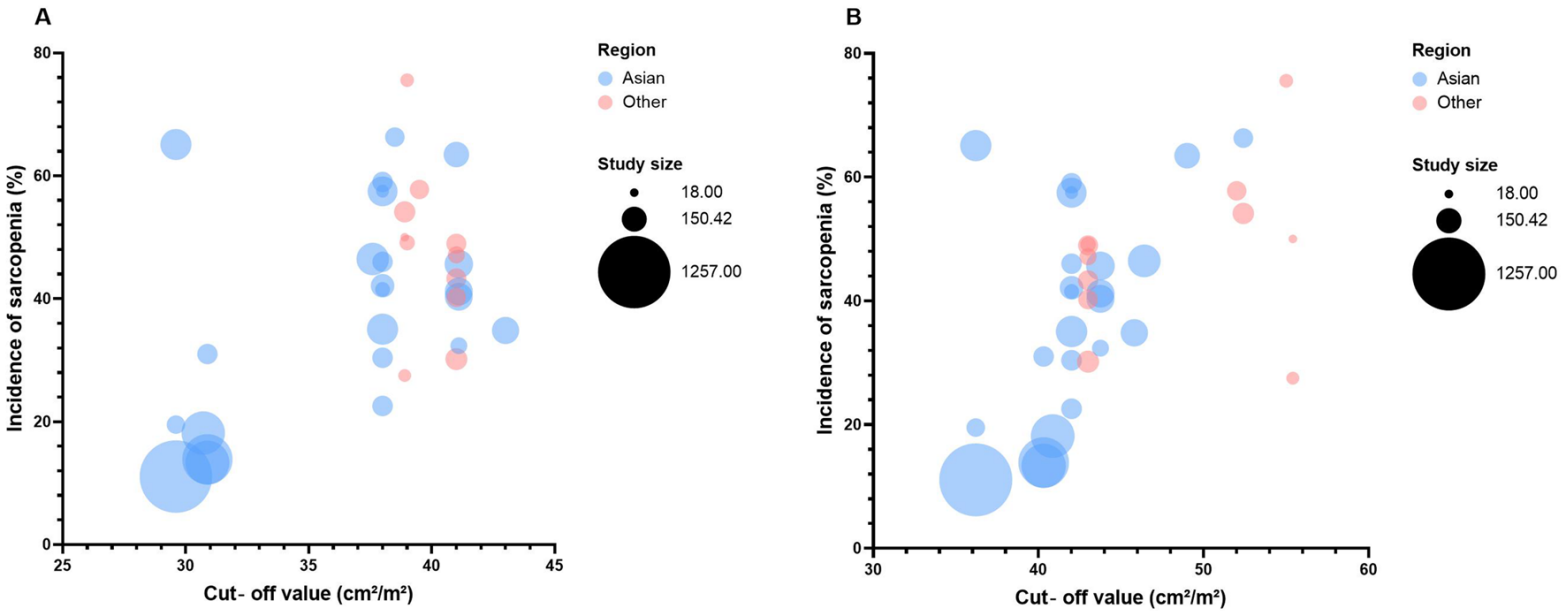
A bubble plot showing the cut-off values of SMI.

A total of 57 studies involving 9790 HCC patients were included in the meta-analysis. The studies were conducted in different regions, with 41 studies^3,24,26–28,32–37,39–41,43–51,54–60,62,63,66–69,71,73–76^ (Japan provided the largest volume of studies, followed by the South Korea) from Asia, and 16 studies^21–23,25,29–31,38,42,52,53,61,64,65,70,72^ conducted in non-Asia regions. The enrolled patients covered all stages of HCC (from BCLC stage 0/A to BCLC stage D) and the corresponding treatments (RFA, hepatectomy, LDLT, TACE, TARE, radiotherapy, sorafenib, lenvatinib, and ICIs). Sarcopenia was diagnosed through CT or (MRI) in all studies. Two studies^3,71^ included only males and three studies identified sarcopenia using the change of muscle mass after or during treatment. Among all included studies, different diagnostic methods and cut‐off values were utilized to identify sarcopenia, with the skeletal mass index (SMI) being the most commonly used method. Figure 2 indicates that the cut-off value of SMI in non-Asia regions tended to be higher than in Asian regions in both sexes. Notably, as the cut-off value increased, the prevalence of sarcopenia increased. The prevalence of sarcopenia ranged from 11.1% to 78.3% in 52 studies available^21,22,24–34,36–52,54–70,72–76^ for the prevalence data, and the pooled prevalence was 41.7% (95% CI 36.2–47.2%) (Fig. S1).

### Overall survival

**Figure 3:**
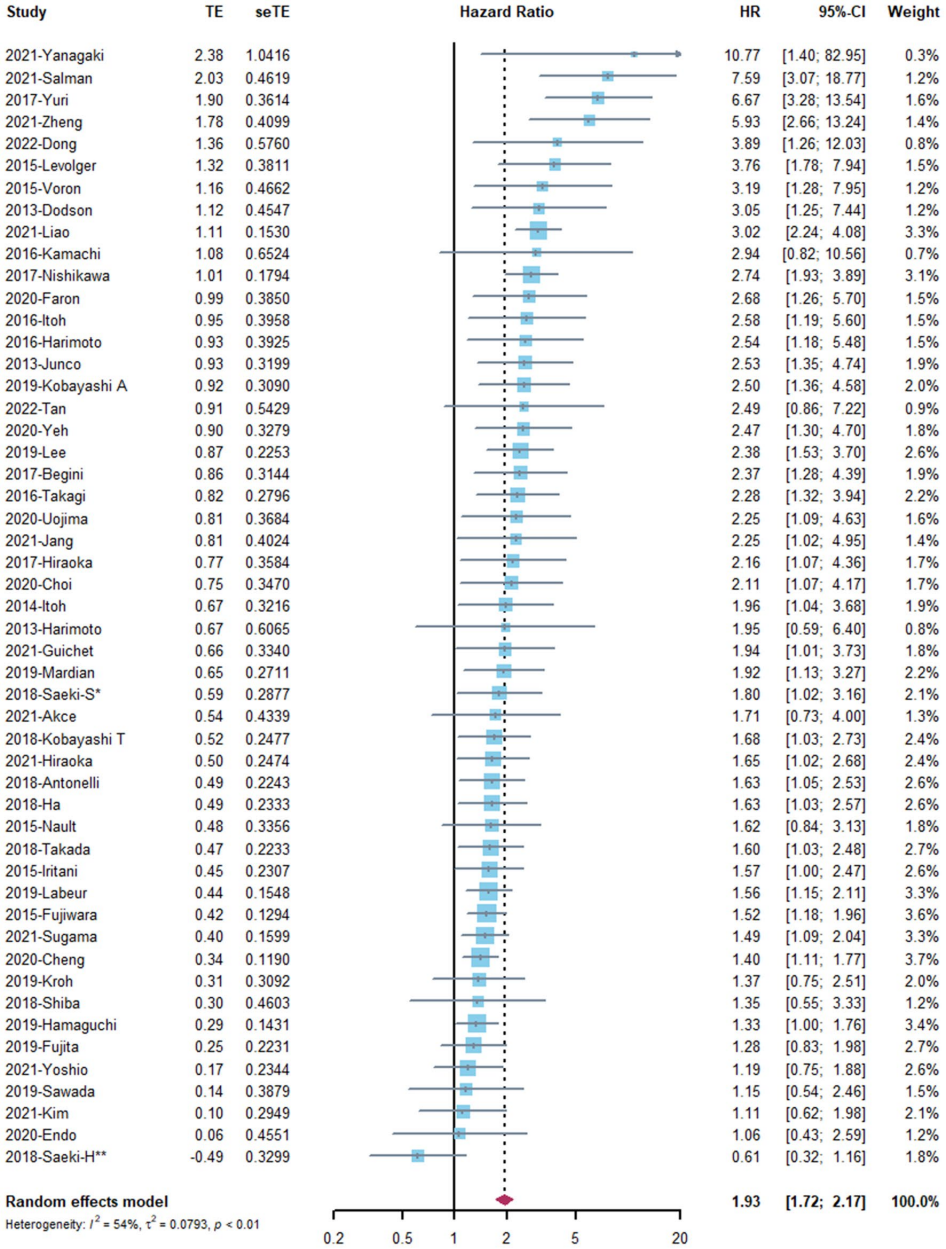
The forest plot of overall survival.

The association between OS and sarcopenia was reported in 51 studies^3,23–34,36,38–43,45–71,73–76^ involving 8768 patients. Considering the high heterogeneity (I^2^ = 54%), a random effects model was used for analysis (Fig. 3). The pooled HR was 1.93 (95% CI 1.73–2.17, *P* < 0.001), which suggested that the presence of sarcopenia was significantly associated with improved mortality. A Baujat plot showed that the study by Liao et al.^69^ significantly contributed to the overall result on both heterogeneity and influence (Fig. S2). Sensitivity analysis was conducted through sequential omission of studies, from which a similar result was obtained (Fig. S3).

### Recurrence

A total of 17 studies^3,24,26,28,31–35,37,44,50,51,67,69,73,74^ involving 3615 patients provided DFS or RFS data. The fixed effects model was used, with the pooled HR (HR: 1.75, 95% CI 1.56–1.96, *P* < 0.001) indicating a higher risk of recurrence in patients with sarcopenia (Fig. S4). Notably, the sensitivity analysis obtained a similar result (Fig. S5).

### Progression‐free survival

Six studies^40,47,57,64,68,76^ involving 581 patients reported the association between PFS and sarcopenia. There was no heterogeneity (I^2^ = 0) in these studies and the fixed effects model showed impaired PFS in patients with sarcopenia, but the trend was not significant (HR: 1.20, 95% CI 0.98–1.48, *P* = 0.082) (Fig. S6).

### Tumor response

Tumor response was evaluated using the modified Response Evaluation Criteria in Solid Tumors (mRECIST) in three studies^40,49,72^, and based on Response Evaluation Criteria in Solid Tumors (RECIST) in another three studies^22,68,76^.

The ORR ranged from 0 to 45.5% in the sarcopenia group and 12.7% to 66.7% in the non-sarcopenia group. The OR was 0.37 (95% CI 0.17–0.81, *P* = 0.012), which indicated that sarcopenia was significantly associated with worse tumor response (Fig. S7). Similar results were obtained after conducting sensitivity analysis (Fig. S8).

Given the high heterogeneity (I^2^ = 57%), a random effects model for analysis was used to pool HR of DCR before the sensitivity analysis. Results showed that there was no significant association between DCR and sarcopenia (OR: 0.56, 95% CI 0.31–1.01, *P* = 0.055) (Fig. S9). In addition, the Baujat plot indicated that the study by Fujita et al.^49^ contributed significantly to heterogeneity (Fig. S10). After it was excluded, the heterogeneity decreased substantially (I^2^ = 14%), with the obtained result (OR: 0.46, 95% CI 0.30–0.69, *P* < 0.001) indicating that sarcopenia was significantly associated with worse disease control (Fig. S11).

### Severe postoperative complications and toxicity of drugs

A total of four studies^24,29,31,52^ reported the rate of severe postoperative complications in two groups (three studies addressed hepatectomy and one study addressed RFA). All postoperative complications were evaluated by Clavien-Dindo classification. The OR was 1.15 (95% CI 0.46–2.88, P = 0.772) and the sensitivity analyses yielded similar findings (Fig. S12, S13). After omitting the study by Levolger et al.^29^, the obtained result (OR: 0.78, 95% CI 0.36–1.67, *P* = 0.519) revealed that sarcopenia was not associated with the occurrences of severe postoperative complications on hepatectomy (Fig. S14).

Drug toxicity data was available in six studies^21,39,40,57,62,76^, of which four studies^21,39,40,57^ addressed sorafenib and two studies^62,76^ addressed lenvatinib. Results indicated that sarcopenia was significantly associated with higher occurrences of severe drug-related adverse events (OR: 2.23, 95% CI 1.17–4.28, *P* = 0.015) (Fig. S15). Moreover, the Baujat plot showed that the study by Mir et al.^21^ contributed significantly to heterogeneity (Fig. S16), and the sensitivity analysis provided similar results (Fig. S17).

### Subgroup analyses

Subgroup analyses of OS were conducted according to the treatments that patients underwent, BCLC stages, diagnostic methods, regions, gender, and the time points of diagnosis. With regard to the seven different treatment methods, results showed that the efficacy of most treatments (six out of the seven) could be influenced by sarcopenia (Table 1). Sarcopenia increased the risk of mortality in most patients who underwent RFA (HR: 4.46, 95% CI 2.64–7.54, *P* < 0.001), but it did not increase the risk of mortality in patients treated with ICIs (HR: 1.27, 95% CI 0.79–2.05, *P* = 0.323). It is worth noting that the earlier the BCLC stage, the higher the risk of sarcopenia. The pooled HR from three studies^56,58,68^ involving 620 advanced HCC patients indicated that sarcopenia may be not associated with the OS of patients at the BCLC C stage (HR: 1.20, 95% CI 0.83–1.75, *P* = 0.331). In addition, subgroup analyses based on the different diagnostic methods, regions, gender, and the time points of diagnosis provided similar results.

**Table 1:** Subgroup analyses of overall survival.

Subgroup	No. of studies	No. of patients	Estimates (HR)	Lower limit to Upper limit	*P*‐value
**Treatment**					
RFA	4	505	4.46	2.64–7.54	< 0.001
Hepatectomy	12	3172	2.03	1.56–2.64	< 0.001
TACE/TARE	6	975	2.23	1.47–3.39	< 0.001
Radiotherapy	2	224	2.13	1.43–3.17	< 0.001
Sorafenib	8	1458	1.69	1.41–2.06	< 0.001
Lenvatinib	4	354	1.80	1.27–2.55	0.001
ICIs	2	159	1.27	0.79–2.05	0.323
**BCLC stage**					
0/A	2	233	4.13	1.38–12.36	0.011
0/A and B	5	660	3.93	2.38–6.50	< 0.001
B and C	13	1715	1.51	1.33–1.72	< 0.001
C	3	620	1.20	0.83–1.75	0.331
**PMI or SMI**					
SMI	33	6727	1.87	1.63–2.13	< 0.001
PMI	8	1115	2.26	1.56–3.28	< 0.001
**Regions**					
Asia	37	7469	1.87	1.64–2.13	< 0.001
Non-Asia	13	1299	2.16	1.73–2.72	< 0.001
**Gender**					
Only males	2	111	1.55	1.15–2.10	0.004
Both	48	8657	1.95	1.73–2.20	< 0.001
Delta or baseline*					
Delta	3	356	2.41	1.34–4.33	0.003
Baseline	47	8412	1.93	1.72–2.16	< 0.001

*ICIs* immune checkpoint inhibitors, *RFA* radiofrequency ablation, *TACE* transarterial chemoembolization, *TARE* transarterial radioembolization, *BCLC* Barcelona Clinic Liver Cancer, *SMI* skeletal muscle index, *PMI* psoas muscle index.

*Delta: sarcopenia was defined with the change of skeleton muscle during or after the treatment.

Furthermore, we evaluated the association between the proportion of patients with different liver diseases and liver functional reserve in every cohort and OS. The results were consistent across all subgroups (Table 2) (Tables S2–S16). Specifically, it was found that the higher the proportion of patients with cirrhosis in a cohort, the more increased the risk of mortality due to sarcopenia (Tables S2–S4). Meanwhile, the lower the proportion of patients with Child–Pugh class A and the higher the proportion of patients with Child–Pugh class B, the more increased the risk of mortality (Tables S13–S16).

**Table 2:** Different liver diseases and liver functional reserve.

Subgroup	Proportion	No. of studies	No. of patients	Estimates (HR)	Lower limit to Upper limit	*P*‐value
**Cirrhosis**						
All cirrhosis	100%	5	641	2.61	1.76–3.86	< 0.001
Proportion > median	62.67–100%	8	1130	2.47	1.77–3.43	< 0.001
Proportion < median	21.58–61.43%	9	2120	1.72	1.46–2.03	< 0.001
**ALD**						
Proportion > median	16.67–47.5%	9	1132	1.53	1.23–1.91	< 0.001
Proportion < median	3.26–15.52%	9	966	1.84	1.50–2.26	< 0.001
**NASH**						
Proportion > median	11.46–100%	5	541	1.67	1.30–2.15	< 0.001
Proportion < median	6.7–10.1%	5	687	1.79	1.44–2.22	< 0.001
**HBV**						
proportion > median	25.1–85.18%	15	2515	1.64	1.36–1.98	< 0.001
proportion < median	6.59–20.18%	18	3662	2.01	1.69–2.37	< 0.001
**HCV**						
Proportion > median	39.19–100%	18	3641	2.07	1.71–2.50	< 0.001
Proportion < median	6.86–38.18%	16	2174	1.78	1.45–2.18	< 0.001
**Child–Pugh class A**						
Proportion > median	80.7–100%	17	3157	1.61	1.44–1.81	< 0.001
Proportion < median	50–78.66%	18	3790	2.27	1.83–2.81	< 0.001
**Child–Pugh class B**						
Proportion > median	22.75–62.67%	16	3550	2.17	1.75–2.69	< 0.001
Proportion < median	2.15–16.67%	15	3056	1.64	1.45–1.84	< 0.001

*AH* alcohol-related liver disease, *NASH* nonalcoholic steatohepatitis, *HBV* hepatitis B Virus, *HCV* hepatitis C Virus.

Subgroup analyses of recurrence were performed based on patients treated with hepatectomy or LDLT. The pooled HR from three studies^3,33,44^ involving 295 HCC patients who underwent LDLT were much higher than the pooled HR from the 12 studies^24,26,31,32,35,37,50,51,67,69,73,74^ on hepatectomy (Table 3).

**Table 3:** Subgroup analyses of recurrence and tumor response.

Subgroup	No. of studies	No. of patients	Estimates (HR/OR)	Lower limit to upper limit	*P*‐value
**DFS/RFS**					
Hepatectomy	12	3155	1.70	1.51–1.92	< 0.001
LDLT	3	295	4.13	2.14–7.97	< 0.001
**ORR**					
Systemic therapy	4	396	0.21	0.09–0.46	< 0.001
TACE	2	265	0.70	0.28–1.72	0.434
**DCR**					
Systemic therapy	4	396	0.52	0.34–0.82	0.004
TACE	2	265	0.55	0.10–3.06	0.500

*LDLT* Living-Donor Liver Transplantation, *TACE* transarterial chemoembolization, *DFS* disease-free survival, *RFS* recurrence-free survival, *ORR* objective response rate, *DCR* disease control rates.

Subgroup analyses of ORR and DCR were conducted on patients who received systemic therapy or TACE. The detailed data of ORR and DCR were available in four studies^22,40,68,76^ on systemic therapy (two studies on lenvatinib, one study on sorafenib, and one study on gemcitabine and oxaliplatin) and two studies^49,72^ on TACE. Results indicated that sarcopenia was associated with lower ORR and DCR in patients that received systemic therapy instead of TACE (Table 3).

### Publication bias

Funnel plots of the OS, recurrence, and PFS provided little indication of asymmetry suggestive of publication bias (Fig. S18A–F). The *P* values from the Egger's test on OS, recurrence, PFS, ORR, DCR, severe postoperative complications, and severe toxicity of drugs were 0.004, 0.024, 0.882, 0.056, 0.277, 0.219, and 0.272, respectively. The Trim and Fill method led to addition of 17 potential unpublished studies (Fig. S19), and the pooled HR of OS was 1.59 (95% CI 1.40–1.81, *P* < 0.001). Similarly, the pooled HR of recurrence was 1.62 (95% CI 1.36–1.93, *P* < 0.001) after five potential unpublished studies were added (Fig.S20).

## Discussion

To date, this study involving 9790 patients is the largest study that has explored the impact of sarcopenia in HCC. Although two previous systematic reviews and meta-analyses^17,18^ described the negative influence of sarcopenia in HCC, they only included 13 studies and 3111 patients, which resulted in the absence of detailed subgroup analyses. The two studies also included patients with other cancers (such as intrahepatic cholangiocarcinoma), which may limit interpretation of their conclusions^77^. In this study, a more comprehensive literature search was conducted, which resulted in more HCC patients being included thereby providing more data for effective subgroup analyses. To determine and decrease the potential heterogeneity, Baujat plots and sensitivity analyses, respectively, were performed to identify the sources of heterogeneity and ensure the stability of obtained results. In addition, to avoid the selection bias and possible calculation error, data was not extracted from the reported Kaplan–Meier curves.

Results demonstrated that sarcopenia was significantly associated with impaired OS, higher risk of tumor recurrence, worse tumor response, and more drug-related adverse events in HCC patients. The calculation results showed that HCC patients with sarcopenia had a 1.93 times higher risk of death, 1.75 times higher risk of recurrence, 0.37 times lower odds of tumor response, and 2.23 times higher odds of adverse drug reactions than HCC patients without sarcopenia. Despite the existence of heterogeneity in the OS data analysis, the sensitivity analyses and the consistency of results derived from different subgroups further validated our results.

In the subgroup analyses, we did not divide the treatment means into curative therapy or palliative treatment like previous meta-analysis^17^ because many studies using curative therapy included patients at the BCLC B or C stage who were beyond the indications of curative therapy^4,24,29,31^. Therefore, the subgroups were clearly divided according to specific therapies and BCLC stages. It was found that patients with sarcopenia at early stage were more vulnerable, and thus we speculated that other risk factors such as tumor metastasis or tumor thrombus were the more important factors leading to death in the patients at more advanced stages. However, the skeletal muscle mass representing systemic nutritional states was associated with the tolerance of operation on the liver like RFA, hepatectomy or LDLT. Meanwhile, HCC patients with sarcopenia suffered higher rates of liver failure, major complications, and intra-abdominal abscess formation^78,79^. Ultimately, OS was significantly impaired in patients with sarcopenia at early stage. In addition, frailty has a very close overlap with sarcopenia^80^. Frailty was associated with an increased risk for mortality and morbidity related to cancer and worse response to treatment^81^, therefore, this could result in decreased numbers of frail patients who received TACE, hepatectomy, or systemic treatment. This selection bias could explain why we found more impact of sarcopenia in the BCLC-0/BCLC-A groups in comparison with those treated with TACE or systemic treatment. Moreover, it could also explain why sarcopenia was not associated with complications after hepatectomy (Fig. S14).

A previous study reported that chronic underlying liver diseases contributed to the process of hepatocarcinogenesis^82^. Moreover, a recent meta-analysis revealed that sarcopenia was highly associated with higher risk of mortality in patients with cirrhosis^6^. Similarly, this study found that HCC patients with more proportion of cirrhosis were at a higher risk of mortality, suggesting the synergistic effect of cirrhosis and HCC. Therefore, more emphasis should be given for the influence of sarcopenia in HCC patients with cirrhosis. In addition, we found that the proportion of patients with different Child–Pugh classes may affect the association between sarcopenia and OS.

Given the impact of sarcopenia on HCC, more attention should be paid on the prevention of sarcopenia and rehabilitation treatment in HCC patients with sarcopenia. Nutritional support and physical exercise are two promising strategies that can improve the skeletal muscle state and long-term prognosis^78^. A previous retrospective study reported that L-carnitine improved sarcopenia progression in HCC patients treated with lenvatinib, and patients with L-carnitine supplementation tended to have a longer median time to treatment failure compared to patients without L-carnitine supplementation^83^. A study conducted in Japan found that in-hospital exercise may prevent sarcopenia in HCC patients who underwent TACE^84^, which suggested the necessity and feasibility of sarcopenia prevention.

However, this meta-analysis had several limitations. First, all the included studies are retrospective studies, which leads to inevitable selection bias and confounding bias. Second, it was hard to compare differences between the hazard of sarcopenia occurring in the course of treatment and the hazard of baseline sarcopenia because only three studies on the change of skeleton muscle mass during or after the treatment were included in the subgroup analyses. Similarly, only two studies explored the association between sarcopenia and prognosis in patients who underwent immunotherapy, thus, it was hard to draw conclusions on the effect of sarcopenia on the efficacy of ICIs. Third, most studies involved patients with different etiologies of chronic liver diseases and Child Pugh classes, thus, it was hard to directly evaluate the impact of chronic liver diseases and liver functional reserve on sarcopenia. This is despite the fact that we divided studies according to the proportion of specific liver disease or Child Pugh class. Fourth, the funnel plots and Egger's test of OS and recurrence implied the presence of publication bias, even though similar results were achieved from the Trim and Fill method (OS: 1.59 vs. 1.93; recurrence: 1.62 vs. 1.75). Fifth, the cut‐off values varied from different diagnostic methods to different research teams. Therefore, meta‐regression analyses should be used to investigate the effect of cut‐off values in future studies. Sixth, to unify the inclusion criteria and facilitate subgroup analyses, we only included studies defining sarcopenia with radiological evaluation. Finally, it should be noted that the level of evidence from this study was rated as low according to GEADE because of the inclusion of retrospective studies which led to inevitable selection bias and confounding bias.

## Conclusion

This meta-analysis demonstrated that sarcopenia was associated with significantly impaired OS, higher risk of tumor recurrence, worse tumor response, and more drug-related adverse events in HCC patients. The presence of cirrhosis and Child Pugh class B increased the hazard of mortality from sarcopenia. HCC patients at early stage of tumor had more impaired OS resulting from sarcopenia than other tumor stages.

## Supplementary Information

Supplementary Tables.

Supplementary Figures.

## Data Availability

The datasets used and/or analysed during the current study available from the corresponding author on reasonable request.
