# Supplementary material for: Association between sarcopenia and clinical outcomes in patients with hepatocellular carcinoma: an updated meta-analysis
Source: Sci Rep. 2023 Jan 17;13:934. doi: 10.1038/s41598-022-27238-z (PMC9845331; doi:10.1038/s41598-022-27238-z)
Supplement: Supplementary file 2 — Supplementary Figures. [file 41598_2022_27238_MOESM2_ESM.pdf]

Supplementary Figure 1 The prevalence of sarcopenia

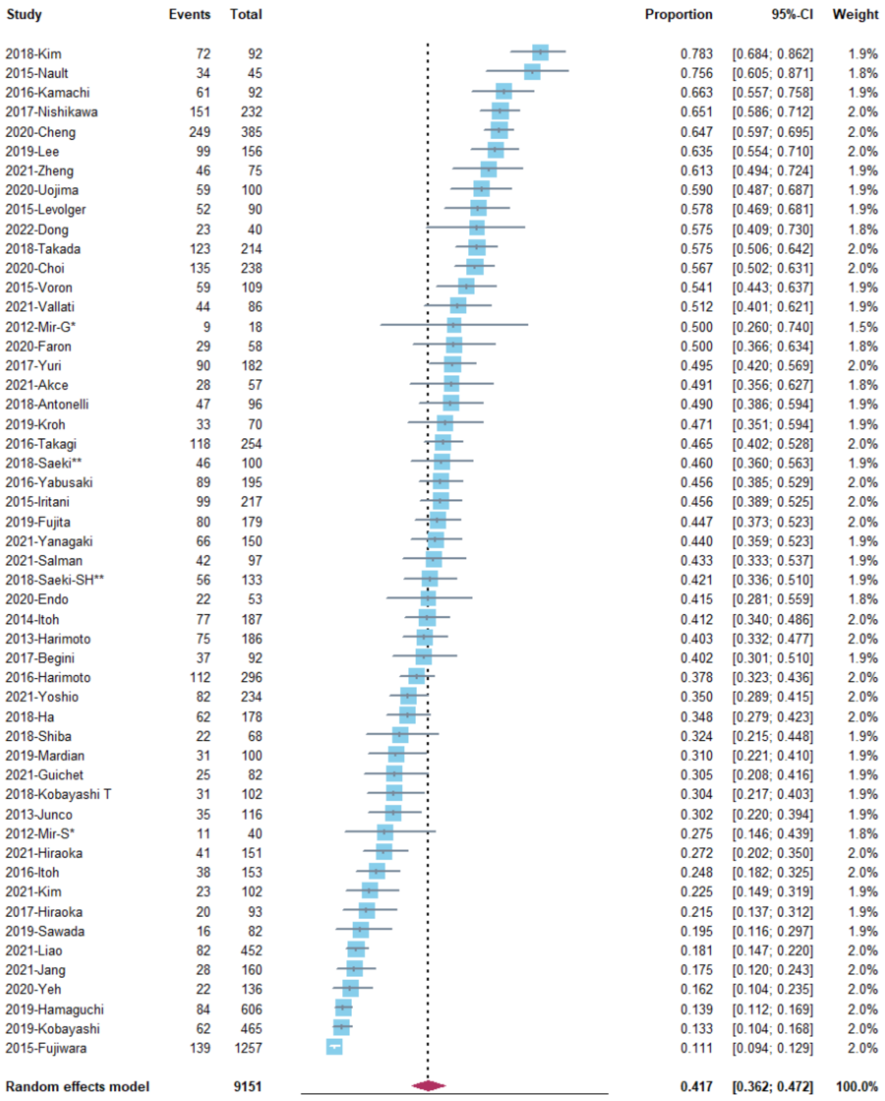

Supplementary Figure 2 The Baujat plot of overall survival

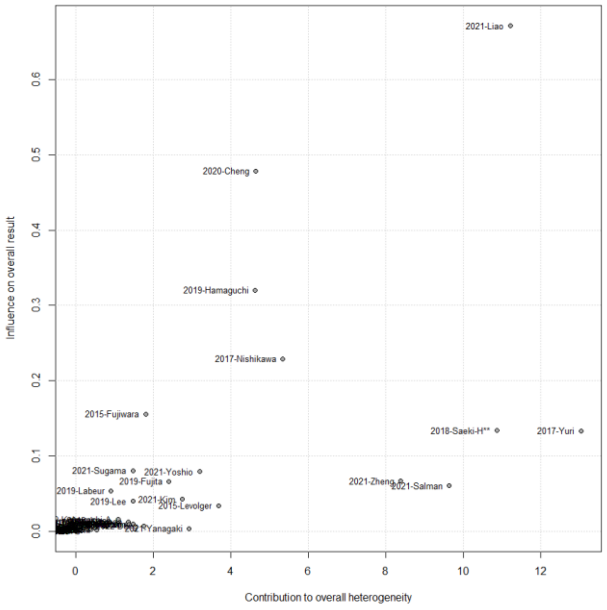

Supplementary Figure 3 The sensitivity analysis of overall survival

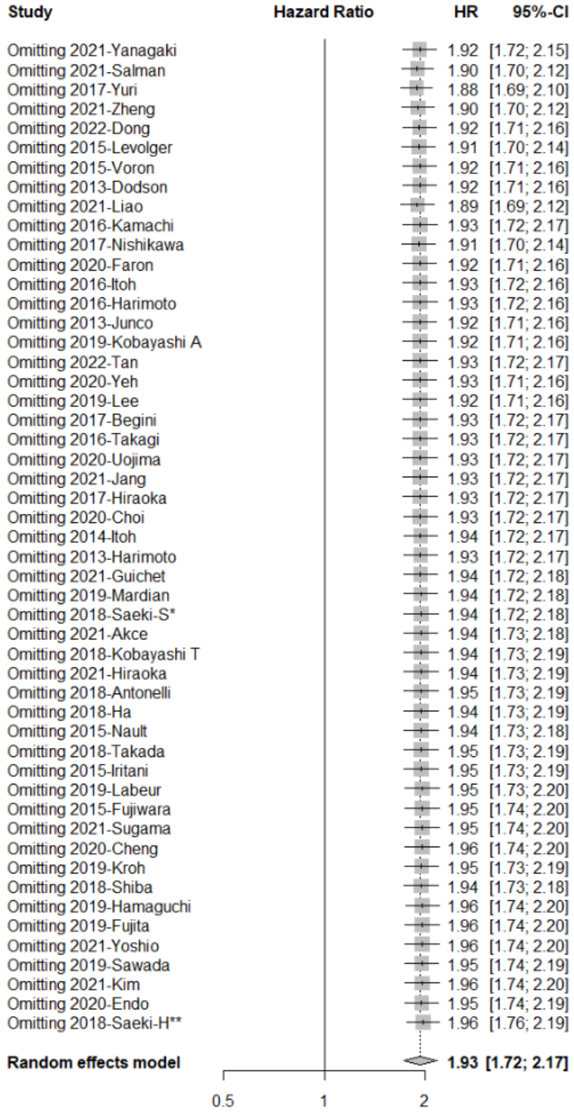

Supplementary Figure 4 The forest plot of recurrence

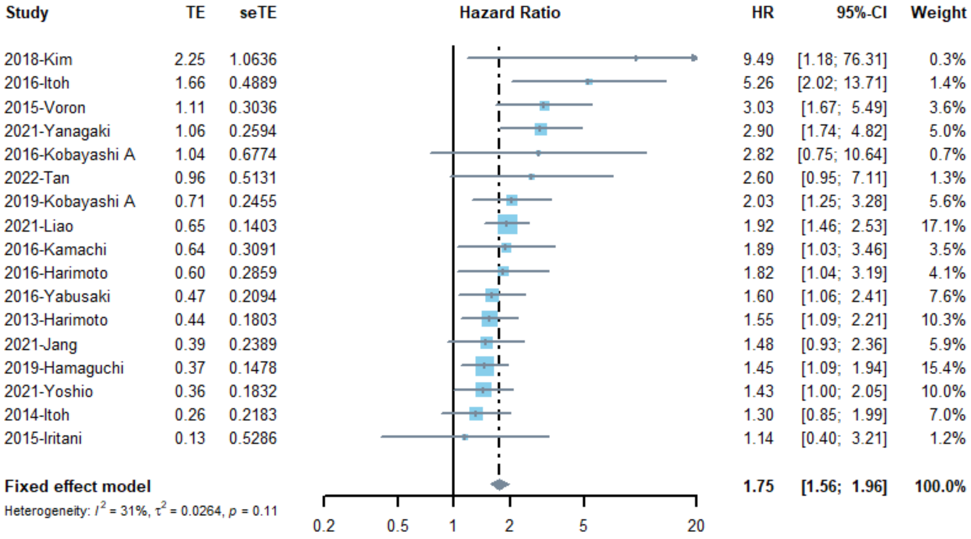

Supplementary Figure 5 The sensitivity analysis of recurrence

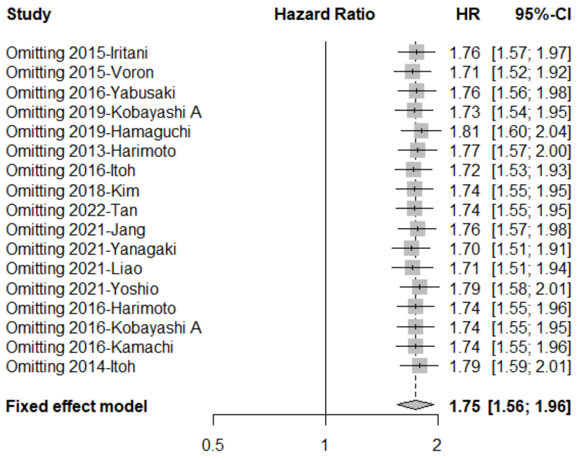

Supplementary Figure 6 The forest plot of progression-free survival

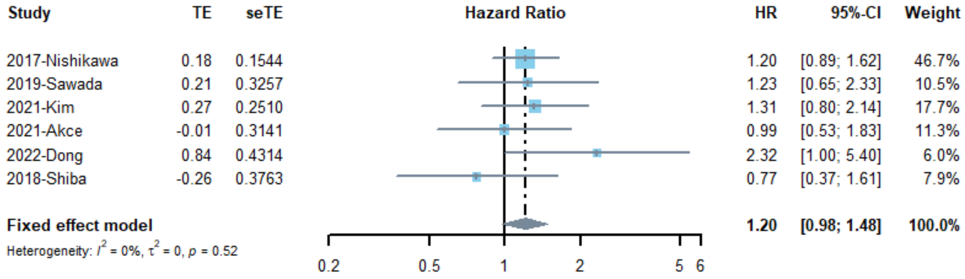

Supplementary Figure 7 The forest plot of objective response rate

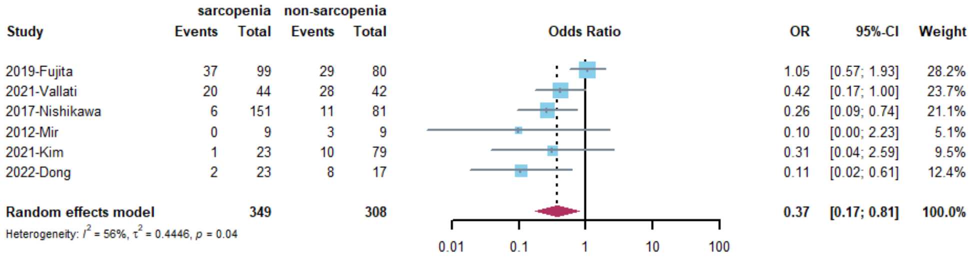

Supplementary Figure 8 The sensitivity analysis of objective response rate

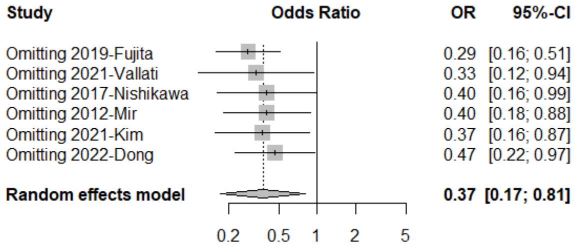

Supplementary Figure 9 The forest plot of disease control rate

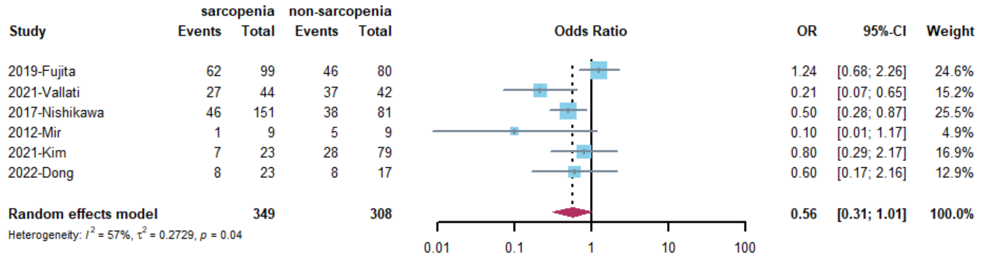

Supplementary Figure 10 The Baujat plot of disease control rate

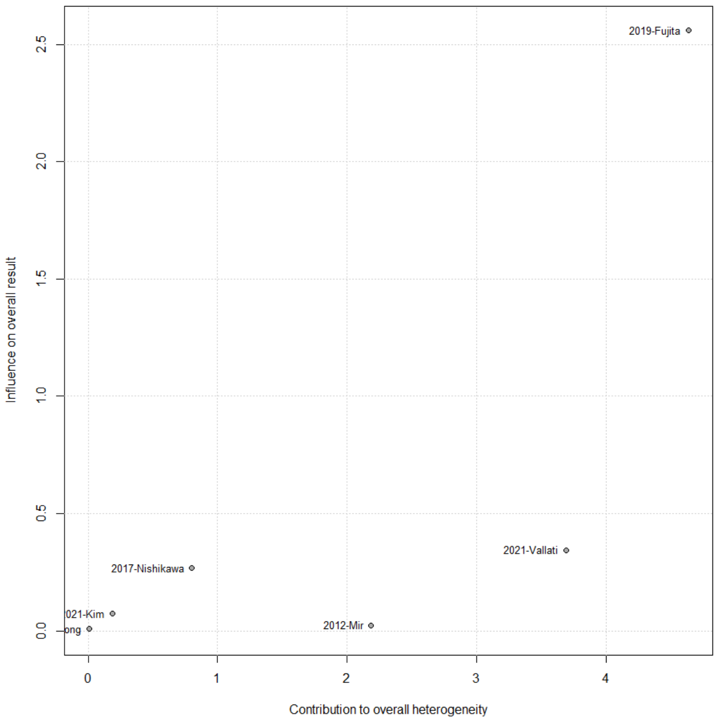

Supplementary Figure 11 The forest plot of disease control rate after omitting the study by Fujita *et al.*

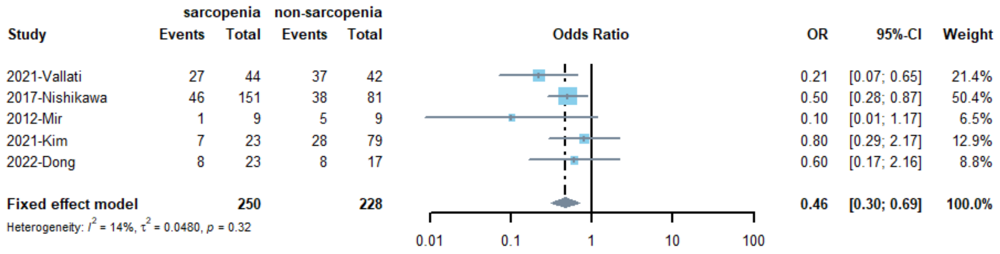

Supplementary Figure 12 The forest plot of severe postoperative complications

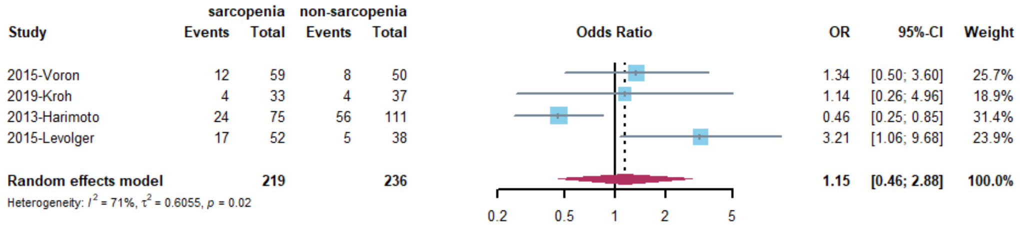

Supplementary Figure 13 The sensitivity analysis of severe postoperative complications

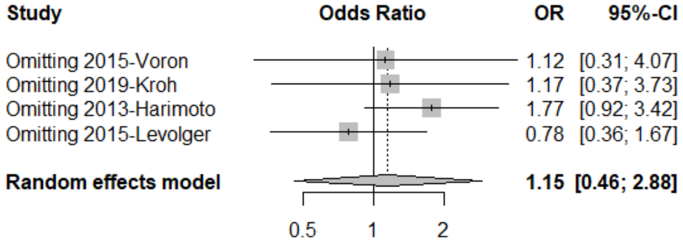

Supplementary Figure 14 The sensitivity analysis of severe postoperative complications on hepatectomy (after omitting the study by Levolger *et al.*)

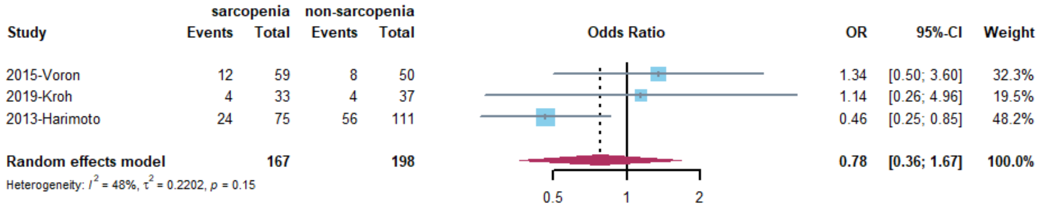

Supplementary Figure 15 The forest plot of severe drug-related adverse events

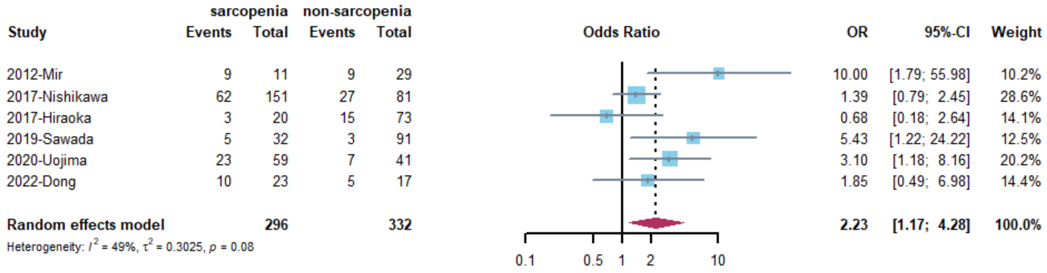

Supplementary Figure 16 The Baujat plot of severe drug-related adverse events

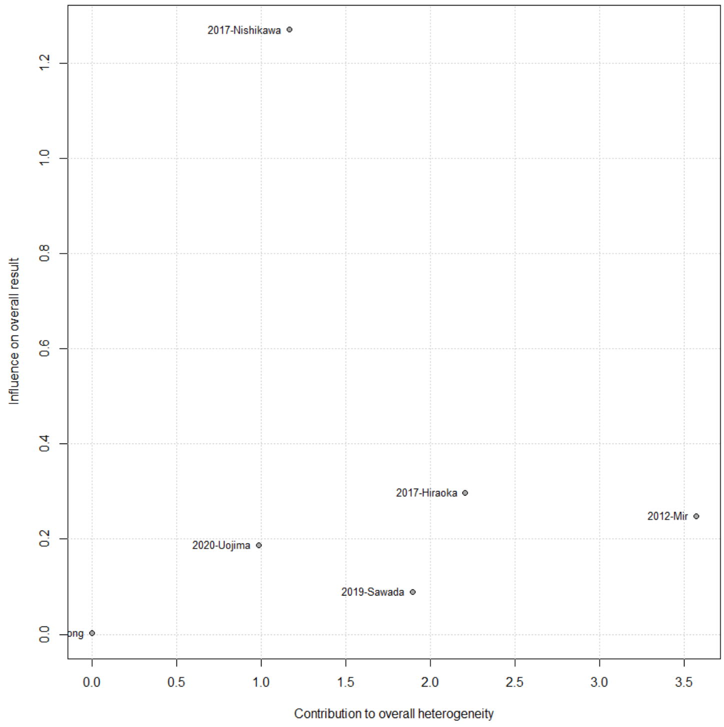

Supplementary Figure 17 The sensitivity analysis of severe drug-related adverse events

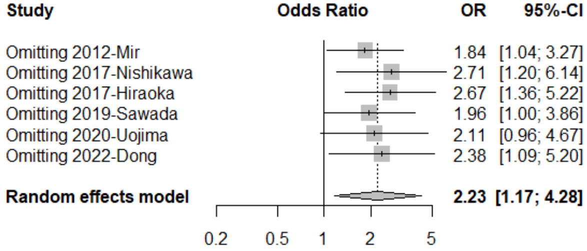

Supplementary Figure 18 Funnel plots of OS (A), recurrence (B), PFS (C), DCR (D), ORR (E), severe drug-related adverse events (F)

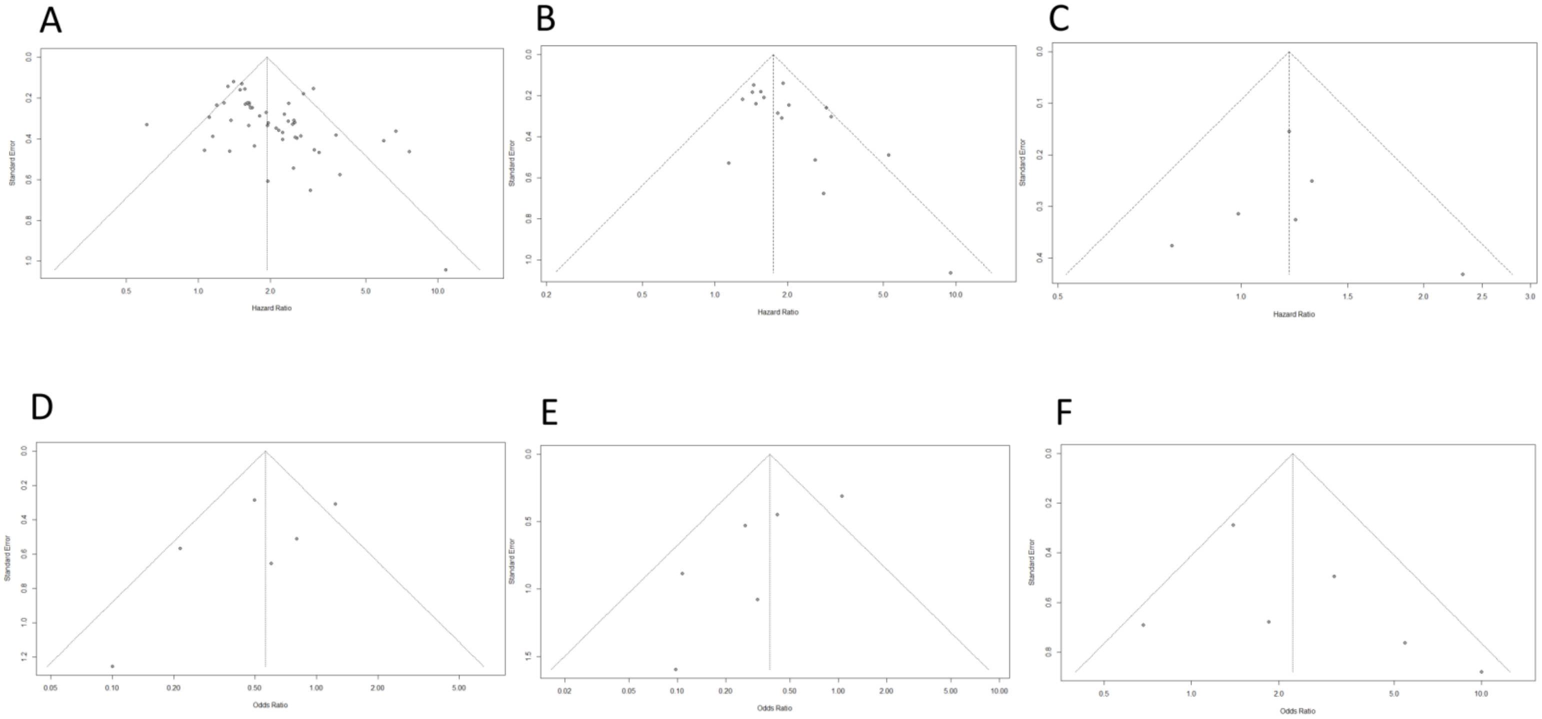

Supplementary Figure 19 The Trim and Fill method: OS

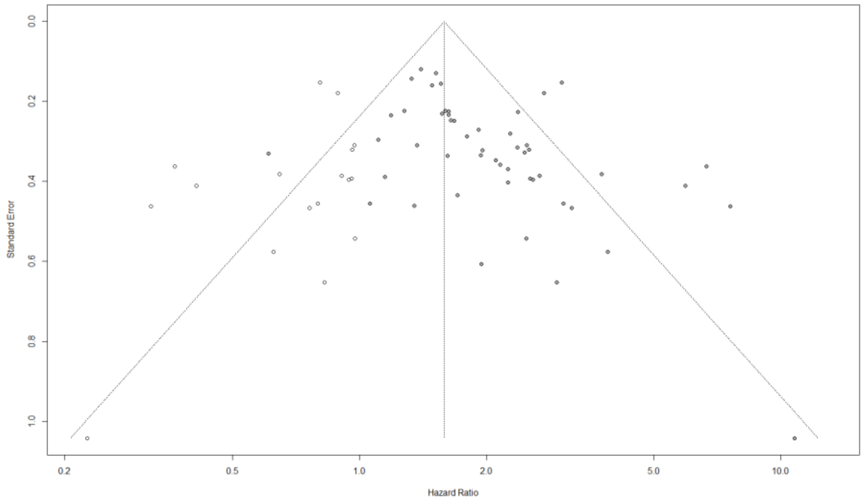

Supplementary Figure 20 The Trim and Fill method: recurrence

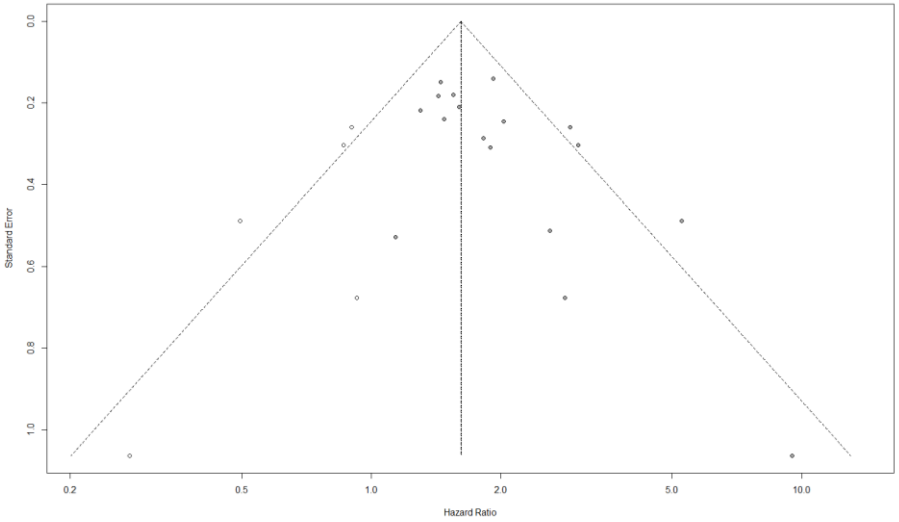

Supplementary Table 1 The characteristics of included studies

Year	Author	Region	Treatment	Diagnostic method	Cut-off value	Stage of HCC	Outcome	number of patients (number of sarcopenia)	Gender (male/female)	Newcastle–Ottawa scale
2012	Olivier Mir	France	sorafenib	SMI	Male:55.4; Female:38.9	BCLC stage C	PFS, OS, toxicity	40 (11)	/	7
2012	Olivier Mir	France	gemcitabine and oxaliplatin as second-line	SMI	Male:55.4; Female:38.9	BCLC stage C	PFS, OS, , tumor response, toxicity	18 (9)	15/3	7
2013	Judith Meza-Junco	Canada	TACE/TARE/ablation/Combined treatment/BSC	SMI	Male, 43 for BMI < 25, 53 for BMI > 25; Female, 41	TMN (I/II/III/IV): 54/38/21/3	OS	116 (35)	98/18	7

2013	N Harimoto	Japan	hepatectomy	SMI	Male:43.75; Female:41.1	TMN (I/II/III/IV): 29/95/49/13	OS, DFS, postoperative complications	186 (75)	145/41	8
2013	Rebecca M Dodson	USA	TACE or TARE	TPA	Male: 477 mm^2/m^2; Female: 338 mm^2/m^2	BCLC stage B and C	OS	109	/	7
2015	Soichi Iritani	Japan	resection/RFA/ TACE or TAI/radiation	FFM	37	Stage (I/II/III/IV):52/71/66/28	OS, DFS	217 (99)	146/71	7
2014	Shinji Itoh	Japan	Hepatic Resection	SMI	Male:43.75; Female:41.1	/	OS, RFS	190 (77)	146/44	7
2015	Naoto Fujiwara	Japan	RFA and TACE	SMI	Male:36.2; Female:29.6	BCLC(0/A/B/C/D): 181/588/427/47/14	OS	1257 (139)	828/429	9
2015	Thibault Voron	France	Hepatectomy	SMI	Male:52.4; Female:38.9	/	OS, DFS, postoperative complications	109 (59)	92/17	8
2015	Stef Levolger	The Netherlands	RFA	SMI	Male:52.0; Female:39.5	BCLC (0/A/B/C): 15/30/36/9	OS, postoperative complications	90 (52)	63/27	8
2015	Jean-Charles Nault	France	Sorafenib/ brivanib: 44/8	SMI	Male:55; Female:39	BCLC stage B and C	OS	45 (34)	/	7

2016	Norimitsu Yabusaki	Japan	hepatic resection	SMI	Male:43.75; Female:41.1	TNM (I/II/III/IVA/IVB): 20/112/42/19/2	RFS	195 (89)	157/38	7
2016	Saori Kamachi	Japan	surgical resection or RFA	SMI	Male:52.4; Female:38.5	BCLC (0/A/B): 29/47/16	OS, RFS	92 (61)	65/27	8
2016	Norifumi Harimoto	Japan	hepatic resection	SMI	the actual skeletal muscle area was 85% smaller than the calculated skeletal muscle area	TNM (I/II/III/IV): 48/156/74/18	OS, DFS	296 (112)	221/75	8
2016	Atsushi Kobayashi	Japan	hepatectomy	Delta PMI	-0.160	TNM (I+II/III+IV): 69/43	RFS	112	90/22	8
2016	Kosei Takagi	Japan	curative hepatectomy	SMI	Male:46.4; Female:37.6	TNM (I/II/III/IV): 28/110/83/33	OS	254 (118)	207/47	7
2016	Shinji Itoh	Japan	LDLT	skeletal muscle mass-to-VFA ratio (SVR)	Lowest quartile	BCLC stage A and B	OS, RFS	153 (38)	86/67	7
2017	Yukihisa Yuri	Japan	RFA	PMI	Male:6.31; Female:3.91	BCLC stage A and B	OS	182 (90)	111/71	7
2017	Paola	Italy	Ablation,	SMI	Male, 43 for BMI	BCLC(A/B/C/D):38/23	OS	92 (37)	62/27	7

	Begini		TACE, hepatic resection, supportive care, orthotopic liver transplant		< 25, 53 for BMI > 25; Female, 41	/26/5				
2017	Hiroki Nishikawa	Japan	sorafenib	SMI	Male:36.2; Female:29.6	Stage (I/II/III/IVA/IVB):1/18/79/46/88	OS, PFS, tumor response, toxicity	232 (151)	181/81	8
2017	Atsushi Hiraoka	Japan	sorafenib	PSI	Male:4.24; Female:2.5	TNM(II/III/IVA/IVB): 10/28/11/44	OS, toxicity	93 (20)	81/12	7
2018	Takamasa Kobayashi	Japan	TACE	SMI and delta SMI	Male:42; Female:38	TNM(I/II/III/IV): 11/22/46/23	OS	102 (31)	70/32	8
2018	Young Ri Kim	Korea	LDLT	PM thickness	12 mm/m	beyond the Milan criteria	RFS	92 (72)	62/30	8
2018	Hitomi Takada	Japan	sorafenib	SMI	Male:42; Female:38	BCLC stage B and C	OS	214 (123)	166/48	8
2018	Issei Saeki	Japan	sorafenib	SMI	Male:42; Female:38	Stage (II-III/IV): 30/70	OS	100 (46)	72/28	9
2018	Issei Saeki*	Japan	sorafenib and HAIC	SMI	Male:42; Female:38	BCLC stage C	OS, tumor response	Sorafenib: 78 (32); HAIC:55 (24)	Sorafenib: 57/21 HAIC: 42/13	7
2018	Yeonjung Ha	Korea	/	SMI	Male:45.8; Female:43	BCLC (0+A/B/C/D): 44/20/100/14	OS	178 (62)	141/37	8

2018	Giulio Antonelli	Italy	sorafenib	SMI	Male, 43 for BMI < 25, 53 for BMI > 25; Female, 41	BCLC stage B and C	OS	96 (47)	75/21	8
2018	Shintaro Shiba	Japan	Carbon Ion Radiotherapy	SMI	Male:43.75; Female:41.10	Stage (I/II/III):57/7/4	OS, PFS, toxicity	68 (22)	41/27	7
2019	Koji Sawada	Japan	sorafenib	SMI	Male:36.2; Female:29.6	BCLC(A/B/C): 8/35/39	OS, PFS, toxicity	82 (16)	67/15	8
2019	Tim A Labeur	The Netherlands	sorafenib	SMI	Male, 43 for BMI < 25, 53 for BMI > 25; Female, 41	BCLC stage B and C	OS, TTP	278	220/58	9
2019	Masashi Fujita	Japan	TACE	PMI and delta PMI	Male:6; Female:3.4	TNM I/II/III/IVA/IVB 8/37/41/9/4(non-sarcopenia); 6/33/30/8/3(sarcopenia	OS, tumor response	179 (80)	130/49	8
2019	Atsushi Kobayashi	Japan	Hepatectomy	SMI	Male:40.31; Female:30.88	TNM(I/II/III/IV): 64/183/147/71	OS, RFS	465 (62)	367/98	9
2019	Yuhei Hamaguchi	Japan	Hepatectomy	SMI	Male:40.31; Female:30.88	TNM (I + II/ III + IV): 361/245	OS, RFS	606 (84)	484/122	9
2019	Yan Mardian	Indonesia	TACE, sorafenib, or supportive Treatment	SMI	Male:40.31; Female:30.88	BCLC(A/B/C/D):7/25/59/9	OS	100 (31)	74/26	8
2019	Joongyo Lee	Korea	Radiotherapy	SMI	Male:49; Female:41	LCSGJ (I/II/III/IVA/IVB):3/1	OS	156 (99)	128/25	8

						5/48/69/21				
2019	Andreas Kroh	Germany	liver resection	SMI	Male, 43 for BMI < 25, 53 for BMI > 25; Female, 41	Milan criteria (beyond/within): 58/12	OS, postoperative complications	70 (33)	49/21	7
2020	Anton Faron	Germany	TARE	FFMA	Male: 3582mm^2; Female: 2301mm^2	BCLC(A/B/C):1/22/35	OS	58 (29)	45/13	7
2020	Tsung-Yi Cheng	Taiwan	After the treatment of sorafenib	TPMT/BH	16.8	BCLC stage C	OS/PPS	385 (249)	302/83	8
2020	Haruki Uojima	Japan	lenvatinib	SMI	Male:42; Female:38	BCLC(B/C):49/51	OS, toxicity	100 (59)	75/25	8
2020	Kei Endo	Japan	lenvatinib	SMI	Male:42; Female:38	BCLC(B/C):19/44	OS	53 (22)	53/10	8
2020	Kanghyug Choi	Korea	Resection/RFA/TACE/systemic therapy:8/38/187/3	PMI	Male:4.98; Female:1.17	BCLC(0/A/B/C):43/95/43/57	OS	238 (135)	193/45	8
2020	Wen-Shuo Yeh	Taiwan	RFA	PMI	Male:4.24; Female:2.5	BCLC stage 0 and A	OS	136 (22)	78/58	8
2021	Atsushi Hiraoka	Japan	lenvatinib	PSI	Male:4.24; Female:2.5	BCLC(A/B/C/D):2/52/96/1	OS, toxicity	151 (41)	116/35	8
2021	Giulio Eugenio	Italy	TARE	delta PMI	decrease in the delta PMI 1 month after	BCLC stage B and C	Tumor response	86 (44)	65/21	8

	Vallati				TARE					
2021	Nalee Kim	Korea	nivolumab	SMI	Male:42; Female:38	BCLC stage C	OS, PFS, tumor response, toxicity	102 (23)	87/15	8
2021	Mehmet Akce	USA	Anti-PD-1 Antibody	SMI	Male:43; Female:39	BCLC(B/C):7/50	OS, PFS	57 (28)	44/13	8
2021	Ahmed Salman	Egypt	RFA	SMI	Male, 43 for BMI < 25, 53 for BMI > 25; Female, 41	BCLC stage A	OS	97 (42)	72/25	8
2021	Hee Yoon Jang	Korea	curative hepatic resection	PMI	Male:3.33; Female:2.38	BCLC(0/A/B/C): 11/107/31/11	OS, RFS	160 (28)	120/40	8
2021	Sachiyo Yoshio	Japan	surgical resection	SMI	Male:42; Female:38	Stage (0/1/2/3/4): 25/ 56/ 60/ 40/ 53	OS, DFS	234 (82)	183/51	8
2021	Chengyu Liao	China	hepatectomy	SMI	Male:40.86; Female:30.71	BCLC(0/A/B/C): 24/274/16/140	OS, RFS	452 (82)	386/66	8
2021	Phillip L Guichet	USA	TARE	FFMA	Male:31.97; Female:28.95	BCLC(A/B/C): 16/29/30	OS	82 (25)	65/17	7
2021	Mitsuru Yanagaki	Japan	Hepatic Resection	area of the psoas muscle	Male:70 cm^2; Female:46.1 cm^2	TNM(I/II/III/IV): 20/75/35/8	OS, DFS	150 (66)	118/32	8
2021	Xiaoming	China	TACE	cross-section	Delta CSA muscle	BCLC(A/B/C): 10/47/18	OS	75 (46)	63/12	7

	Zheng			area (CSA) of paraspinal muscles						
2021	Yusuke Sugama	Japan	TACE/HAIC/sorafenib/ lenvatinib: 14/38/17/18	PMI	TACE/HAIC: 4.98 for males sorafenib/ lenvatinib: 5.66 for males and 4.61 for females	BCLC(A/B/C/D):29/39/18/1	OS	87	61/26	7
2022	Yifei Tan	China	LDLT	PMI	6.25	/	OS, RFS	50	Male	6
2022	Dong Dong	China	lenvatinib	SMI	Male:42; Female:38	BCLC(B/C):12/28	OS, PFS, tumor response,	40 (23)	37/3	7

HCC: hepatocellular carcinoma; OS: overall survival; DFS: disease-free survival; RFS: recurrence-free survival; PFS: progression-free survival; TTP: time-to-progression; RFA: radiofrequency ablation; TACE: transarterial chemoembolization; TAI: transcatheter arterial infusion; TARE: transarterial radioembolization; BCLC: Barcelona Clinic Liver Cancer; LDLT: Living-Donor Liver Transplantation; BSC: Best supportive care; TPA: total psoas muscle area; FFM: fat-free mass; SMI: skeletal muscle index; PMI: psoas muscle index; FFMA: fat-free muscle area; TPMT/BH: the value of transverse psoas muscle thickness per body height; PSI: psoas muscle area index.

The group of sorafenib in this study was abbreviated as "2018-Saeki-S", and the group of HAIC in this study was abbreviated as "2018-Saeki-H**".
